# Implementing Blended Care to Discontinue Benzodiazepine Receptor Agonist Use for Insomnia: Process Evaluation of a Pragmatic Cluster Randomized Controlled Trial

**DOI:** 10.2196/43738

**Published:** 2023-04-07

**Authors:** Kristien Coteur, Marc Van Nuland, Birgitte Schoenmakers, Sibyl Anthierens, Kris Van den Broeck

**Affiliations:** 1 Academic Centre for General Practice Department of Public Health and Primary Care KU Leuven Leuven Belgium; 2 Department of Family Medicine and Population Health University of Antwerp Antwerp Belgium

**Keywords:** benzodiazepines, long-term use, deprescriptions, deprescribing, telemedicine, general practice, insomnia, cognitive behavioral therapy, eHealth

## Abstract

**Background:**

Long-term use of benzodiazepine receptor agonists (BZRAs) remains common despite European guidelines advising that these drugs be used in the lowest possible dose and for the shortest possible duration. Half of all BZRAs are prescribed in family practice. This creates a window of opportunity for discontinuation in primary care. Therefore, the effectiveness of blended care for the discontinuation of long-term BZRA use in adult primary care patients with chronic insomnia disorder was tested in a multicenter, pragmatic, and cluster randomized controlled superiority trial in Belgium. In the literature, information on implementing blended care in a primary care setting is scarce.

**Objective:**

The study aimed to contribute to a framework for the successful implementation of blended care in a primary care setting by increasing our understanding of this complex intervention through an evaluation of e-tool use and views and ideas of participants in a BZRA discontinuation trial.

**Methods:**

Based on a theoretical framework, this study evaluated the processes of recruitment, delivery, and response using 4 components: a survey on recruitment (n=76), semistructured in-depth interviews with patients (n=18), web-based asynchronous focus groups with general practitioners (GPs; n=19), and usage data of the web-based tool. Quantitative data were analyzed descriptively, and qualitative data were analyzed thematically.

**Results:**

For recruitment, the most common barriers were refusal by the patient and the lack of digital literacy, while facilitators were starting the conversation and the curiosity of patients. The delivery of the intervention to the patients was diverse, ranging from GPs who never informed the patient about their access to the e-tool to GPs consulting the e-tool in between consultations to have discussion points when the patient visited. Concerning response, patients’ and GPs’ narratives also showed much variety. For some GPs, daily practice changed because they received more positive reactions than expected and felt empowered to talk more often about BZRA discontinuation. Conversely, some GPs reported no changes in practice or among patients. In general, patients found follow-up by an expert to be the most important component in blended care, whereas GPs deemed the intrinsic motivation of patients to be the key element of success. An important barrier to implementation by the GP was time.

**Conclusions:**

Overall, the participants who had used the e-tool were positive about its structure and content. Nevertheless, many patients desired a more tailored application with feedback from an expert and personal tapering schedules. Strict pragmatic implementation of blended care seems to only reach GPs with an interest in digitalization. Although not superior to usual care, blended care could be a complementary tool that allows tailoring the discontinuation process to the personal style of the GP and the needs of the patient.

**Trial Registration:**

ClinicalTrials.gov NCT03937180; https://clinicaltrials.gov/ct2/show/NCT03937180

## Introduction

### Long-term Benzodiazepine Receptor Agonist Use

Psychotropic substances that may pose a threat to public health or cause social problems are monitored by the International Narcotics Control Board, by tracking the licit movement of these substances worldwide. Among these substances are benzodiazepine receptor agonists (BZRAs), which are often used in the treatment of anxiety and insomnia. Although multiple expert groups promote a reticent policy for their use, the International Narcotics Control Board’s yearly reports show that BZRA consumption is, and remains, remarkably high in Belgium [1].

Long-term use remains common in clinical practice, mostly as continuous use of a low, steady dosage, despite European guidelines advising BZRAs to be used in the lowest possible dose and for the shortest possible duration [2-7]. Since half of all BZRAs are prescribed in family practice, there is a window of opportunity for discontinuation in primary care [8].

### Tapering BZRA Use

To reduce BZRA use, gradual tapering is the gold standard [9]. Its effectiveness can be increased by adding a nonpharmacological intervention, such as psychotherapy, self-help instructions, or patient education [10,11]. Providing such a combined intervention can be time-consuming in practice. Therefore, blended care holds much promise. In blended care, an interactive educational e-tool is combined with face-to-face clinical consultations with the care provider [12]. This approach has proven to be successful in the treatment of multiple psychiatric and somatic conditions, including sleeping disorders and substance use disorders [13-15]. Therefore, we aimed to establish an evidence-based blended care approach for the discontinuation of chronic BZRA use in adult primary care patients with chronic insomnia disorder using a multicenter, pragmatic, and cluster randomized controlled superiority trial (registered at ClinicalTrials.gov NCT03937180) [16].

In preparation for this trial, we noticed a gap in the literature regarding the implementation of blended care for deprescribing in a primary care setting. Thus, a multicomponent process evaluation was set up to increase our understanding of this complex intervention. We report on both the setup and results of this process evaluation to inform future implementation projects on blended care in general practice.

## Methods

### Study Design

**Figure 1:**
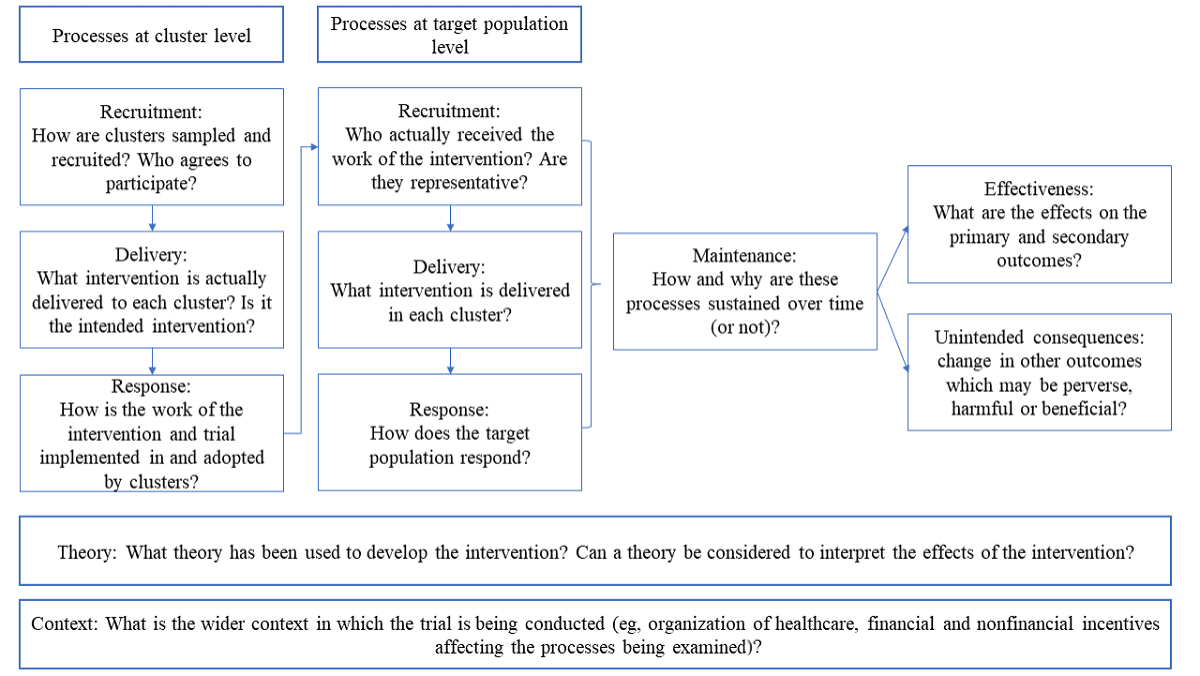
Framework model for designing process evaluations of cluster-randomized controlled trials by Grant et al [17].

Process evaluations clarify how the intervention and implementation can be improved by mapping important influences, depending upon the context in which the intervention is used [17]. To evaluate the implementation of blended care, this study was prespecified and nested in a cluster randomized controlled trial (c-RCT) that aimed to test the effectiveness of blended care for the discontinuation of long-term BZRA use in patients with primary insomnia. This was a highly pragmatic trial, in which blended care was implemented without a protocol. More information on the trial design and intervention is provided in Multimedia Appendix 1 [9-16,18-21] and in previous publications [16,18]. Inspired by the previous process evaluations of complex interventions [22,23], a setup based on the framework of Grant et al [17] was created. This framework covers processes involving the clusters as well as the target population, theoretical influences, and contextual factors [17]. With regard to clusters and target population, three specific processes are distinguished: (1) recruitment, (2) delivery of the intervention, and (3) response (Figure 1). The study has been reported following the Revised Standards for Quality Improvement Reporting Excellence 2.0 checklist.

### Recruitment

General practitioners (GPs) received an email invitation to participate in different components of this process evaluation. If necessary, this was followed by multiple reminders or a personal telephone invitation. For the survey, all GPs who had started recruiting patients were invited. For focus groups, the sample was limited to GPs allocated to the intervention group. For the interviews, the patient sample was purposively selected to obtain variation in age, sex, discontinuation status, and the intensity of the use of the e-tool. Patients were first invited by their GP. If interested, they could either connect with the researchers themselves or ask to be contacted.

### Data Collection

#### Before the Trial

Before the trial, a feasibility study of GPs from different regions in Belgium was conducted. The aim was to assess the possibilities for patient recruitment in a pragmatic general practice trial. During a structured interview, led by an external expert from a clinical contract research organization, the process and expected outcomes of recruitment were discussed, as well as the research activities in the trial protocol.

#### Before the Intervention

During the trial, but before the intervention started, patient recruitment was tracked by the GPs with screening logs, documenting any reasons for exclusion.

#### During the Intervention

Usage data of the e-tool were tracked using Matomo, an open-source web analytics program, showing us the number of unique page views during a visit. These data were retrieved from July 5, 2019, when the e-tool was launched, until December 7, 2020, when the last patient in the intervention group reached week 26, and their access to the e-tool ended.

Moreover, when all clusters were randomized, a survey on patient recruitment was conducted. It explored GPs’ actions and views using 14 questions, focusing on workload, preparation, patient selection, and the perception of patient views.

#### After the Intervention

Both GPs’ and patients’ views and ideas about the implementation of blended care were explored using qualitative methods. To prevent intervention bias in the c-RCT, this was performed after all patients had passed the 26 weeks’ time point. Data from the perspective of GPs were collected in 3 asynchronous web-based focus groups using a dedicated tool called FocusGroupIt. This method was preferred because of the geographical spread of the participating GPs and their availability during the COVID-19 pandemic. Two focus groups were conducted in Dutch, with 10 and 15 GPs invited, respectively, resulting in 7 respondents in each group. One focus group was conducted in French, with 17 GPs invited, resulting in 5 respondents. Researcher KC monitored them and intermittently discussed with the team (MVN, SA, and KVdB). The researchers could prompt GPs to provide more information. All data were collected between October 2020 and February 2021, with a maximum duration of 2 months per focus group. Data from the perspective of patients were collected through semistructured in-depth interviews. Interviews were conducted in Dutch or French by native speakers, namely KC and a research assistant Magali Le Clef, between September 2020 and March 2021. All interviews were audiotaped. For both the focus groups and the interviews, a prespecified topic guide was used, which is summarized in Textbox 1. The French translation was provided by the French interviewer, which was then back-translated by the Dutch interviewer. Sociodemographic data of the participants in this phase are presented in Table S1 in Multimedia Appendix 1.

**Topic list for interviews with patients and general practitioners (GPs).**

**Individual interviews with patients (n=18)**

- Background: insomnia and medication use
- Blended care:
  Interaction with the GP
  Differences with usual care
  e-Tool: satisfaction, advantages, and disadvantages
  Personal motivation and outcome
- Motivation to participate
- Influence of the COVID-19 pandemic crisis

**Asynchronous focus groups with GPs (n=19)**

- Motivation to conduct the trial
- Blended care:
  Previous experience
  Differences with usual care
  e-Tool: integration in their consult, advantages, and disadvantages
  Relation with the patient
- Motivation of patients to participate
- Influence of the COVID-19 pandemic crisis

### Analysis

All data collected on the web, such as usage data, the recruitment questionnaire, and asynchronous focus groups, were exported to Excel (Office 365; Microsoft Corporation) for analysis. Patient interviews were transcribed verbatim.

For quantitative data analysis, descriptive statistics and graphical analysis (using Excel) were used. Qualitative data were analyzed thematically, with a combination of deductive and inductive coding. An initial coding tree was developed based on the interview themes and research questions in the protocol. This was used by 2 independent coders, KC and research assistant Shani De Coster, to process all data in NVivo 12 (QSR International) [24]. When the data did not fit the predefined codes and themes, the coders developed additional codes. Disagreements were resolved through discussion. Moreover, the ongoing analysis was discussed among KC, Shani De Coster, MVN, SA, and KVdB on 5 occasions throughout the data collection and analysis process: after 3, 8, 11, 17, and 18 interviews. Patient interview data were found to be adequate after coding 15 interviews. At this point, no new categories were developed nor were there elements that assigned new meaning to the data. Interviews that were already scheduled at this time proceeded as planned and were included in the analysis.

**Figure 2:**
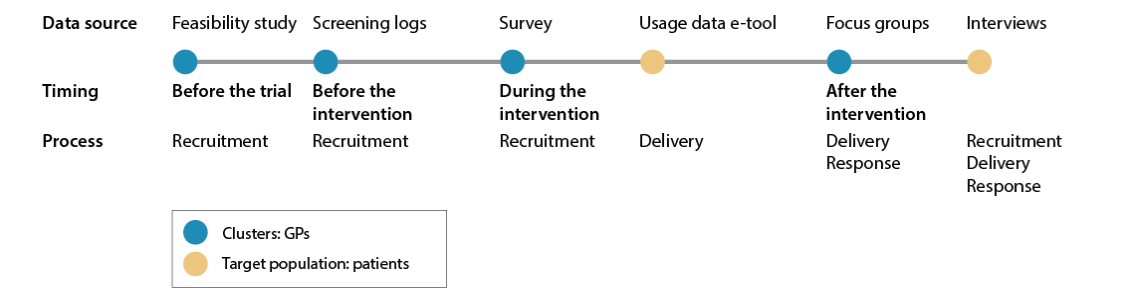
Flowchart of data collection with timing and sample for each method and link to the processes that were discussed. GP: general practitioner.

Finally, all available data were integrated when analyzing the results per process. For example, the results with regard to delivery of the intervention come from 3 data sources: usage data of the e-tool, focus groups with the GPs, and interviews with the patients. A visual representation of the data sources for each process is presented in Figure 2.

### Ethics Approval and Informed Consent

The c-RCT was approved by the Ethics Committee for Research of Universitaire Ziekenhuizen Leuven, Katholieke Universiteit Leuven (UZ/KU Leuven; ref S61194). This process evaluation, nested in the c-RCT, was approved by the Ethics Committee for Research of UZ/KU Leuven (ref S63790) on June 15, 2020. Written informed consent was obtained from all participants before data collection.

## Results

### Recruitment

#### Clusters

All GPs with whom we discussed the setup of the trial during the feasibility trajectory responded positively. Although it was mentioned that recruitment of patients could be challenging owing to the digital components in this trial, it was deemed possible. When commencing the trial, 11 live training sessions on usual care for the discontinuation of BZRAs were organized across the country. When attending this training, GPs were invited to participate in the trial. Furthermore, GPs were recruited through the universities’ network and personal contact. GPs who had not attended a live training session on usual care were offered a self-study package. Reasons for refusal of participation were not documented at this stage. In total, 121 GPs started recruiting patients. These GPs were located across Belgium in both the Dutch (Flanders, Brussels) and French regions (Wallonia, Brussels).

Procedures for patient recruitment and randomization were previously described in a protocol paper [16]. Following the reviewers’ feedback and careful consideration by the funder and trial management team, the initial intracluster correlation coefficient of 0.11 was corrected to 0.04. This led to a decrease of the minimally required number of patients per cluster from 10 to 5. To prevent unnecessary prolongation of the trial, a deadline for the recruitment of patients was established in December 2019. GPs who could not recruit a minimum of 5 patients did not continue to the phase of randomization. Finally, 99 GPs were randomized with the following profile: 62 Dutch GPs and 37 French GPs—86 working in group practices, of which 20 were in a multidisciplinary group practice and 19 in a community health center. Most GPs were not familiar with blended care before participating in the trial. Some mentioned that they had prior experience referring their patients to books, podcasts, or a web-based journal but did not define this as blended care.

#### Target Population

GPs prepared for the recruitment of patients in different ways. Half of the respondents (39/76, 51%) confirmed in the survey that they have used an audit of their electronic patient records to determine whether they treated a sufficient number of eligible patients before committing to the trial. Once confirmed, the GPs were advised to solicit all eligible patients. However, 36% (27/76) of participants confirmed that they did not invite specific patients on purpose because they expected a negative reaction. Although multiple strategies for recruitment were offered, the most commonly used strategy was to address patients during routine consultations.

Between May 23 and December 20, 2019, a total of 1814 patients were screened, of which half (n=898, 49.5%) were ineligible or declined participation. The main reason for exclusion was refusal by the patient. Patients’ interest was influenced by the way the study was explained and their fear of having to discontinue their medication, according to almost half of the GPs (37/76, 49%). Roughly one-third of the GPs (23/76, 30%) also indicated that the subject of the trial, BZRAs and sleep, kept patients from participating. Another important reason for not participating was a lack of computer skills, according to 80% (61/76) of GPs. The lack of digital literacy was also the second most common reason for exclusion in the screening logs.

According to the interviews, patients mainly participated because their GP asked. They felt that they were the right candidate or grateful to be able to do something good for their GP. Some patients explicitly wanted support in discontinuing their medication and trying new methods. Others were eager to learn about sleep, whereas for still others, curiosity about scientific trials or the effects of tapering down were the most motivating.

In total, 916 patients participated. Most patients were female (653/916, 71.3%), with an average age of 61 (SD 11.5) years. These characteristics are typically associated with long-term BZRA use, as described in scientific literature [9,25]. Multimorbidity was registered in 65.7% (602/916) of patients. BZRA use was limited to 1 product for most patients (675/916, 73.7%) and a moderate dose to treat insomnia (an equivalent of 10.5 mg diazepam on average, SD 8.9). For approximately 12.9% (118/916) of patients, 2 products were registered, and for a few patients, 3 or 4 products were registered (Table S2 in Multimedia Appendix 1).

### Delivery

#### Clusters

After randomization, GPs in the intervention group were informed about their access to the interactive e-tool. They were reminded that they were free to implement the intervention as they deemed appropriate. Although it was available, some GPs did not explore the e-tool nor use it in practice. Others threw a glance at it but did not process the modules thoroughly. The most common reason for this was time deficit. A third group of GPs went through all modules, and one specifically mentioned reviewing them in between consultations.

#### Target Population

At the start of the intervention, GPs had to inform the patients about their allocation outcome and access to the e-tool. Although there was an explicit guideline to do so, not all GPs passed on this information. Nevertheless, the e-tool was available to all patients in blended care, and after having completed their web-based questionnaires, patients were automatically referred to its home page. The implementation of the intervention, which consisted of using the e-tool and personal consultations with the GP, varied strongly. Both the motivation of the patients and the follow-up provided by the GP were found to be key factors.

For some GPs, the use of the e-tool was strongly linked to the intrinsic motivation of patients. It was also mentioned that, in blended care, patients were supposed to take responsibility for their health, with the role of the GP being reframed to support the patient in their individual trajectory. Some were able to take on this role by introducing the e-tool and showing the patients around beforehand by reviewing the sleeping journal that was completed by the patient or by providing feedback and follow-up of any questions that the patient may have had. In addition, scheduling time to talk about the patient’s BZRA use was viewed as a positive opportunity: “I liked the idea of a separate consultation for benzo use, because otherwise this often doesn’t happen in a consultation with many other questions” (GP6). For other GPs, it was difficult to obtain and keep patients motivated; work with the tools provided, such as the sleeping journal; and build a meaningful conversation around the topic. Another GP felt insufficiently involved in the intervention, as they were not sure about what content the patients had processed: “The intervention happens largely over the head of the GP. You have little insight into what he or she sees” (GP9).

This high variability was confirmed in the patients’ narratives. Some GPs were very absent and did not follow up with the patient:

I haven’t actually had anything to do with the doctor at all. They just sent me everything online, that’s all they did?

Patient 10

Whereas others provided so much information that the e-tool was considered abundant by the patient:

My self-help module was name GP. I think name GP secretly gave the necessary information over the phone.

Patient 1

Moreover, some patients did not know that they had access to web-based information, whereas others regularly repeated the web-based exercises. Some patients also described that they would complete their questionnaires because these were obligatory for the trial but did not move on to the information modules. Patients who did view the modules were mostly triggered by curiosity, eagerness to learn, or extrinsically by the explicit request of their GP:

What she asked me to fill in I will have done.

Patient 5

So they had spoken of that website, he said it might be good to go through it once to see how and what...It might be useful too, and it was useful too.

Patient 12

Patients mostly processed the content of the e-tool without intensely using it. Some read everything once, whereas others revisited interesting modules. Only a few patients mentioned using it daily, mainly to complete the sleeping journal:

The journal that I used to do every day. I liked it because it helped me really tell how things were going.

Patient 18

The daily use of the sleeping journal was also shown by the usage data of the e-tool. The sleeping journal was part of module 1, which has been visited 5398 times. The second most viewed module, with 2797 visits, focused on information about the different types of BZRA, their positive and negative effects, tolerance, and dependency. This was followed by a module on how to stop the use of BZRA, resilience and balance, and tapering and motivation. The least-visited modules focused on myths and habits. (Table 1).

**Table 1:** Chapters in the e-tool sorted by the number of unique page views between July 5, 2019, and December 7, 2020.

Chapters in the e-tool: content description	Unique page views, n
Chapter 1: reviewing your use of sleep medication, including self-examination and sleeping journal	5398
Chapter 2: more information on sleep medication, about the different types of medication, positive and negative effects, tolerance, and dependency	2797
Chapter 6: let’s get to work: stopping sleep medication, about resilience and balance, tapering and motivation	1929
Chapter 5: better sleep without the aid of sleep medication, about sleep hygiene, helping thoughts, and stimulus control	1573
Chapter 3: the truth about sleep medication, about myths and possible solutions for better sleep	848
Chapter 4: do you have good sleeping habits? including a self-examination exercise	820
Extra resources, including more information on sleep patterns and quality	99

### Response

#### Clusters

The major change in clinical practice that GPs described was that they more often start a conversation about discontinuation. One GP also transferred the relevant skills and knowledge to treat other medication dependencies:

Blended care through teleconsultation etc...is going to be important in the future for addictions of all kinds. I use the folder and motivational techniques a little more now also with other addictions.

GP8

For some patients, the prescription policy was changed, meaning that they would no longer systematically renew prescriptions. “Yes, I think that, thanks to the study, our discourse has been modified, BZRAs are no longer systematically prescribed, and the question of withdrawal is more systematically asked” (GP12). For others, the value of their restrictive policy was confirmed: “It confirmed our policy to prescribe benzos very restrictively, only for a short course of treatment” (GP11). Then again, for others, nothing changed: “Not changed, already had tapering schedules” (GP13). Finally, 1 GP confirmed that working with the sleeping journal was very useful to learn more about the patient’s situation, although it was also time consuming.

Time was introduced as an important barrier to the implementation of blended care. GPs mentioned that they would need more time to learn how to work with the program, that an integration in their electronic health record software would help them save time, and that they lacked time to explain the potential benefits of blended care to the patient. Although the potential relevance of digital interventions was recognized, learning to work with blended care was perceived as an assignment:

The general practitioner is busy. If there is no accreditation in return, every assignment will be seen as lacking in priority. That’s just the way it is...Using this kind of media together WITH the patient, is not yet mastered by us, GPs. That culture may change over time. Presently it is left to the techie colleagues, I suspect. Interesting concept nonetheless!

GP4

#### Target Population

Not all GPs noticed changes among their patients, be it in motivation or actual BZRA reduction. However, when they did, this change was associated with the patient being sufficiently informed. GPs described that informed patients had an increased awareness of the harmful side effects of their BZRA use and a more critical attitude toward BZRA intake. In this group, some patients discontinued BZRA use completely, although dose reductions were more common:

I was amazed at how well the majority of patients responded to the modules, with awareness of the danger of Z drugs. They didn’t stop, but most at least reduced the dose.

GP10

Informed patients also raised the topic themselves in consultations; needed less information from the GP, which led to more time for shared decision-making; and were deemed easier to motivate toward discontinuation.

A recurring view among GPs was that patients had to be intrinsically motivated to not only use but also benefit from the interactive e-tool:

Blended care generally does not work very well in practice, patients take little time for it. The highly motivated patients do have support from it, find additional information. For the motivated patient, I think it helps in practice. But for patients who are not motivated, it has no added value.

GP5

In contrast, patients considered contact with the GP and feedback to be more important elements:

I find that combination very positive. It means that you are not alone, that you have some follow-up. That makes the commitment a little bigger.

Patient 2, Dutch

A good relationship with the GP also meant that patients were not afraid to ask questions and discuss problems. Furthermore, patients who expressed no interest in using an e-tool were willing to have an extra consultation with the GP to discuss their BZRA intake if they were invited: “Yes, if the GP asked to visit sometime that would be more effective than tools that I have to click on the computer” (Patient 13). Another patient remarked that the initiative could come from both sides:

She never brought it up, but neither did I. I might as well have said doctor don’t we want to see what we can do about it?

Patient 11

Finally, some patients mentioned that follow-up could also be performed by other professionals, as long as they are experts in the topic.

Other factors that influenced patients to use the information modules were accessibility, referring to the log-in procedure that must be kept as simple as possible; free availability; and time. Future use of the interactive e-tool was deemed interesting in case of problems such as relapsing after discontinuation:

I would love to have that because if should you ever have a relapse, which can always happen, you can motivate yourself again to it [discontinuation]. That it remains accessible.

Patient 17

Overall, patients responded positively to the content of the information modules. The medication education and sleep hygiene modules were the most appreciated. Some patients also referred to the example tapering schedule but would have preferred a tailored version. Finally, the patients responded ambiguously to the sleeping journal. For some, it helped to obtain more insight into their sleep problems, whereas others became anxious by completing it:

Like a sleep diary, I didn’t use that because I thought I’m going to get obsessed with my sleep here...That you’re going to worry even more because you’re going to worry even more about. Am I sleeping well, or am I lacking sleep, I can’t get to sleep? That’s such a circle.

Patient 3

### Other Elements

#### Maintenance

During the trial, multiple GPs had to end their participation prematurely. For most, this was caused by an increase in workload (see the *Context* section) or a decline in health. Furthermore, the main problem expressed by the GPs was insufficient time to discuss blended care and to motivate patients. Nevertheless, multiple GPs acknowledged that blended care will most likely be part of future care models owing to the upcoming of telemedicine. Participating GPs suggested that for a sustainable implementation of blended care, an integration in their medical software is essential. This would increase both the reach and use of the intervention. Accessibility was very important to keep GPs motivated. Finally, this intervention could also benefit from patient empowerment to increase bilateral exchange regarding medication use.

#### Effectiveness

In this c-RCT, blended care was not superior to usual care for the discontinuation of long-term BZRA use in the treatment of insomnia, in general practice. In the intervention group, 18% (82/456) of patients discontinued BZRA use. In the control group, 19.8% (91/460) of patients discontinued BZRA use. There was no statistically significant difference found for any of the outcomes [18].

#### Unintended Consequences

No unintended outcomes of the intervention were identified during this process evaluation.

### Theory

The e-tool was developed based on the transtheoretical model of behavior change [26] and the Behaviour Change Wheel (BCW) [27]. Furthermore, its content was based on evidence-based methods for the discontinuation and nonpharmacological treatment of insomnia. First, tapering is the gold standard for discontinuing long-term BZRA use. Previous research has shown that tapering combined with cognitive behavioral therapy is more likely to yield better results [28]. Therefore, modules on medication education were included: an exercise on the benefits and risks of continuing BZRA use, an example tapering schedule, and the possibility of adding a tailored tapering schedule. Second, cognitive behavioral therapy for insomnia (CBT-I) is the gold standard for treating insomnia without medication [29]. It combines psychoeducation with both cognitive and behavioral interventions. In addition, when delivered digitally, CBT-I is found to be efficacious in reducing insomnia symptoms [15]. Moreover, previous studies have described a positive association with self-reported discontinuation of sleep medication use [30,31]. Therefore, CBT-I was the basis for the modules on sleep and for many of the exercises in the web-based tool.

As the discussed methods are also key elements in providing optimal usual care, the hypothesized difference between intervention and control in this trial was in the time investment by the GP and potential motivational effects in the patient to start discontinuation.

### Context

The start of the intervention depended on the flow of recruitment, randomization, and the GP scheduling the baseline visit. All blended care trajectories started between September 5, 2019, and June 7, 2020. In March 2020, the COVID-19 pandemic hit Belgium, and the first restrictions were set. With regard to the impact of the pandemic on the trial, opposing views were described. For most GPs, the pandemic heavily limited the implementation of the intervention because of a severe shift in treatment priorities, which resulted in less time to discuss the ideas and views of patients:

Too much influence. I wanted to offer more guidance. In the beginning, I invited the patients on my afternoon off so that I could really start the journey with them. Covid took away my free time.

GP8

In addition, fewer face-to-face consultations took place, which decreased the opportunities to discuss BZRA use:

It was difficult to monitor patients’ progress during the pandemic. Prescriptions were given by telephone for a large part of the time.

GP9

Patients confirmed that they avoided personal contact with the GP:

No especially during the COVID-19 period, if I need a prescription now I send an email and go and get it from the secretariat. I don’t see the doctor. I try to see the doctor as little as possible.

Patient 16

Then again, some GPs believed that the pandemic had no impact at all because they followed up their patients with alternative methods:

No [influence of COVID-19]. Patients were followed up by mail. Consultations in practice were no more or less supportive than without covid.

GP13

For many patients, the pandemic introduced new types of stress, especially by interfering with their work-life balance and the care for their children (in young families), limiting their social contacts (mainly mentioned by older patients), and concern for their own or loved ones’ health. For some, this resulted in worsening of their sleep problems and, sometimes, an increase in BZRA intake. For others, it brought more peace and calm, creating the ideal moment to discontinue their BZRA use:

[Changes] rather in the positive sense than in the negative sense. Actually at the beginning of corona I was home a lot because I had a lot of overtime and I had to take it. For me that was a good opportunity to take less sleep medication though, that’s also when I stopped. I knew then I was going to be able to sleep in, so if I don’t sleep at night, I have the peace of mind of I can sleep in, I can sleep longer.

Patient 6

## Discussion

### Principal Findings

This study describes the evaluation of a pragmatic implementation of blended care in general practice for the discontinuation of long-term BZRA use for insomnia using process data from multiple sources, including the perspective of GPs and patients. Complexity in this trial seemed to be driven by diversity. There was diversity in the participants’ profile, the delivery, and response to the intervention, which were associated with the consultation style of the GP, as well as reactions and assertiveness among patients. For each good practice that was discussed, an opposite narrative could be found in the data.

GPs deemed the intrinsic motivation of patients to be the key element of success in blended care. However, patients generally considered personal follow-up as the most important component. Although not all participants saw value in blended care, some benefits were repeatedly described, such as an increase in dialogue because patients were more informed and more patients reduced their dose. However, for many GPs, navigating this approach does not come naturally and was therefore interpreted as an extra assignment. Finally, although many patients were excluded because of a lack of digital skills, owing to digitalization and the upcoming of telemedicine, blended care was perceived by multiple participants as part of future general practice.

### Strengths and Limitations

This process evaluation provides insight into the needs of GPs and patients to implement blended care for deprescribing in a primary care setting. It was conducted in Belgium, which strengthened the study because of the country’s cultural diversity, with a Dutch and French primary care culture and a European metropolis, Brussels. This diversity was captured as best as possible by purposively selecting patients for the interviews. Besides interviews, we used asynchronous web-based focus groups, which have been found to generate the equivalent of high-quality ideas as in-person focus groups but in a more cost-effective manner [32,33]. Furthermore, this study was built on a theoretical framework that made us consider elements that would otherwise not have been captured, for example, the broader context. Finally, we focused on this evaluation of the blended care intervention, which allowed for an in-depth exploration of the processes of delivery and response. Had we prioritized a better understanding of the trial, it would have been important to set up a similar exploration in the control group. This study has several limitations that must be considered. First, there was some sampling bias in the trial related to the need for basic e-literacy skills because the largest proportion of long-term BZRA users is known to be older adult women [34]. Therefore, a cautious interpretation of the results is warranted. Second, the feasibility study was not included in the protocol for this process evaluation nor was it separately reviewed by an ethics committee. Nonetheless, it is included in this study report after careful consideration because it holds information on the initial perception of the trial by GPs. Third, the recruitment process was not extensively questioned in the focus groups or interviews. It could have been possible to verify the findings from the survey, but the researchers decided that, with the limited resources available, the processes of delivery and response were more important for understanding the intervention. Fourth, usage data were not collected at the individual level, which means that we could not evaluate intervention fidelity. Page visits were captured using clicks. As Boots et al [35] previously discussed, clicks represent visiting a page but do not represent processing the content of that page. Therefore, the participants were specifically questioned about the useful and irrelevant elements of the e-tool during the interviews. More detailed information about the proportion of patients and how intensely they used the e-tool could have contributed to contextualizing the results of the trial. Finally, the pragmatic character of the trial revealed the importance of the GP’s consultation style and preferences when implementing a novel intervention.

### Future Outlook

Several factors are to be considered for the successful implementation of blended care to discontinue long-term BZRA use in a primary care setting. First, this study revealed contradictory views of GPs and patients with regard to motivation for action. According to GPs, the intrinsic motivation of the patient was considered to be an important factor for success. The same view was recently described in a paper on health care professionals’ perspectives on integrated blended care [36]. This finding contrasts with patients indicating that requests by the GP motivated them to participate and process web-based information. Nevertheless, both findings concur with the BCW, which places motivation and opportunity at the core of behavior change, together with a patient’s capabilities [27]. Furthermore, the explicit request of a GP to a patient could be viewed as empowerment, which influences a patient’s belief about their own capabilities, according to the Theoretical Domains Framework (TDF) [37]. Both behavioral change frameworks are relevant for the design and implementation of future interventions (Figure S3 in Multimedia Appendix 1).

Second, GPs acknowledged the importance of patient education but missed the link with motivation. Informed patients were described as being more aware of the side effects of BZRA use, which relate to a change in *beliefs about consequences*. The latter is one of the domains in the TDF that has been associated with the concept of reflective motivation in the BCW [19]. GPs also described alterations in the conversation about BZRA use and patients becoming more critical toward their BZRA intake because they were better informed. In the TDF, this relates to *beliefs about capabilities*, which also influences reflective motivation and which can be strengthened by patient empowerment [19]. Furthermore, patients described that a good relationship with the GP was important to enable discussion of problems. In addition, communication with the GP assured patients that they were not going through the process alone, which was more stimulating. GPs indirectly also requested more bidirectional communication, as they felt insufficiently involved in the intervention and were unaware of the information that patients had processed.

Third, 36% (27/76) of GPs acknowledged that they did not test their own assumptions with regard to the willingness of patients to discuss BZRA use (by not mentioning the trial to specific eligible patients). This is a barrier to patient-centered care, as this study revealed that the way GPs communicated about the trial strongly influenced patients’ willingness to participate, that multiple GPs were positively surprised about their patients’ responses when starting the conversation about discontinuation, and that follow-up was an external motivator for patients to use the e-tool. Therefore, communication skills are vital to qualitative care, as is emphasized by several patient-physician communication experts [38-40].

Fourth, the findings of this study imply that we should strive toward more self-management of chronic insomnia by patients. This could be done by reframing the role of the health care provider as one of the support pillars in the patient’s individual trajectory. Although this was the view of some participating GPs, recent research shows that others might struggle with this change in role from therapist to coach [36]. According to patients, follow-up by an expert was important, including feedback when using the e-tool and personal tapering schedules. Follow-up could be provided by anyone, given that they had proper knowledge. This has been confirmed by a recent review showing that brief interventions with other health care providers, such as pharmacists, are also successful in discontinuing BZRAs [9]. Finally, the importance of support and encouragement cannot be underestimated, as it was also associated with successful discontinuation in the EMPOWER study, in which a deprescribing brochure on benzodiazepines was mailed to older adult patients [41].

Fifth, blended care was implemented pragmatically, that is, without a protocol. As there was much diversity in the delivery of the intervention, we questioned the need for guidelines with good practice examples to achieve a more tailored application in clinical practice. On the one hand, a recent study on integrated blended care concluded that matching treatment to the patient’s personal preferences improves implementation in a primary care setting [42]. On the other hand, having to decide when and how to follow the treatment protocol was an important barrier for health care professionals in the same study [36]. Similarly, mixed reactions to blended care among GPs were common in this project, with recognition of digitalization in health care on the one hand and perceiving the intervention as a burdensome task on the other hand. Although this could be related to the context of the COVID-19 pandemic, it could also refer to a luddite culture in general practice and mask the need for training of GPs. Altogether, the lack of effectiveness in comparison to usual care and these mixed reactions provides no reason to invest in nationwide implementation of the intervention in its current form.

Nevertheless, this evaluation has shown that positive effects were attributed to the use of the e-tool. This makes us hypothesize that the wide diversity in delivery and response reflects the diversity in primary care and the complexity in patient profiles of long-term BZRA users. Therefore, future trials should investigate whether complementary complex interventions are needed to address BZRA overconsumption in primary care. If so, interventions could be tested in noninferiority trials, in which superiority testing can still complement the noninferiority test. Additionally, more knowledge of the impact of feedback loops in digital interventions would be highly relevant for future blended care projects.

### Conclusions

The implementation of blended care depends on the preferences of both GPs and patients. Success stories were mainly described by GPs with an interest in digitalization. Patients’ narratives showed that the relationship between the care provider and patient, with communication at its core, was very important for successful implementation. Overall, participants who had used the e-tool were positive about its structure and content. Nevertheless, many patients desired a more tailored application with feedback from an expert and personal tapering schedules. Although not superior to usual care, blended care could be of added value in clinical practice as a complementary complex intervention that allows tailoring of the discontinuation process to the personal style of the GP and the needs of the patient.

## Appendix

Multimedia Appendix 1 Additional information about the trial design, intervention, participants, Capability-Opportunity-Motivation Behaviour model and Theoretical Domains Framework (TDF), and questionnaire and interview guides.

Multimedia Appendix 2 SQUIRE 2.0 (Standards for QUality Improvement Reporting Excellence) checklist.

## Abbreviations

BCW: Behaviour Change Wheel
BZRA: benzodiazepine receptor agonist
CBT-I: cognitive behavioral therapy for insomnia
c-RCT: cluster randomized controlled trial
GP: general practitioner
TDF: Theoretical Domains Framework
UZ/KU: Universitaire Ziekenhuizen, Katholieke Universiteit
