# Supplementary material for: Implementing Blended Care to Discontinue Benzodiazepine Receptor Agonist Use for Insomnia: Process Evaluation of a Pragmatic Cluster Randomized Controlled Trial
Source: JMIR Form Res. 2023 Apr 7;7:e43738. doi: 10.2196/43738 (PMC10132004; doi:10.2196/43738)
Supplement: Multimedia Appendix 1 [file formative_v7i1e43738_app1.pdf]

Multimedia appendix

1. Trial design

A pragmatic cluster randomized controlled trial (c-RCT) was conducted in general practice in Belgium. General practitioners (GPs) were recruited to test the effectiveness of blended care for the discontinuation of BZRAs in patients with sleeping problems. Patients were eligible for participation if they had used BZRAs for minimally six months at 80% or more of the days for primary insomnia, had no severe psychiatric or neurological comorbidities, or contra-indications for discontinuation. Lack of basic digital literacy, operationalized as "working with e-mail and Google to perform an information search", presence of substance use disorders, and not having their patient record managed by the GP, led to exclusion. Cluster randomization was used to avoid contamination bias. Each GP surgery represented a cluster. As the intervention could not be delivered in a blinded set-up, treatment allocation was only possible after sufficient patients consented to participate within each cluster[16]. To increase external validity, a pragmatic set-up was chosen.[20,21] The intervention was compared to optimal usual care, as all participating GPs received a refresher course on the discontinuation of BZRAs. No additional skills training was provided because adoption of the program was meant to be tested in usual circumstances. Furthermore, there was no protocol for the implementation of blended care. Primary outcome of the trial was discontinuation at 52 weeks as assessed by toxicological screening of urine. Secondary outcomes were discontinuation at 26 weeks, quality of life, self-reported discontinuation, and the number of defined daily doses as registered by the GP[16]. Data was collected at baseline, weeks 6, 12, 26 and 52 (Figure 1). All participating GPs were remunerated pro rata.

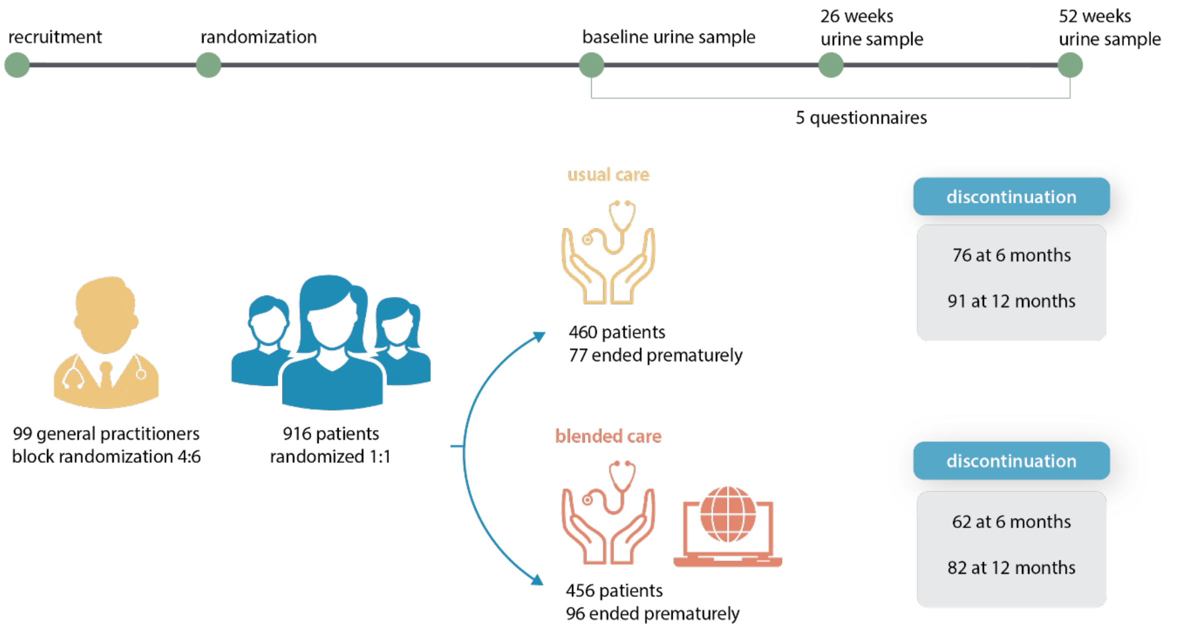

Figure 1. Visual representation of study flow, randomization outcome, and main results

2. Intervention

Blended care consisted of usual care provided by the GP combined with an interactive e-tool. Patients were expected to consult the GP minimally once in the first six months of the trial, which corresponds to minimal standard care when using BZRAs long-term. All other follow-up depended on the needs of both the patient and GP. The interactive e-tool was developed by a multidisciplinary committee of GPs and psychologists. Its aim was to motivate and support patients in discontinuing their use of BZRA, while adapting non-pharmacological remedies for insomnia. It consisted of the following components: (1) a sleeping diary, (2) a tapering schedule, (3) six modules which provide medication education and psychoeducation, focusing on how to improve sleep, and (4) a library with additional links and information. To access the educational modules, patients first had to evaluate their motivation for discontinuing BZRA use with a short survey, and open the sleeping diary. Based on their stage of motivation, the e-tool suggested them a customized trajectory. However, in accordance with self-management principles, patients decided themselves how and when to use the e-tool. Self-management was furthermore enhanced through many exercises with cognitive behavioral techniques, and the possibility of sharing their answers or outcome of exercises with their GP.

Participants in the intervention group had access to the e-tool for 26 weeks, starting at the date of their baseline visit. Participants in the control group had no access to the e-tool during the trial, but were equally given access to the e-tool for 26 weeks when the trial was finished in their cluster.

2A. Detailed structure of the interactive e-tool

An interactive e-tool, named *Slaapzorgeloos* in Dutch and *Dormir Sereinement* in French was offered to participants as a secured website, with adaptability to mobile screens.

HOME PAGE

i. Welcome (back) message
ii. Menu to choose a section of the e-tool
    1. Chapter 1. Reviewing your use of hypnotics
    2. Chapter 2. More information on hypnotics
    3. Chapter 3. The truth about hypnotics
    4. Chapter 4. Do you have good sleeping habits?
    5. Chapter 5. Towards a better sleep without hypnotics
    6. Chapter 6. Get to work: discontinuing use of hypnotics
    7. My sleeping diary
    8. My tapering schedule
    9. Library

Chapter 1
Self-test to evaluate motivation
Sleeping journal
Graphs visualizing sleeping journal entries

Chapter 2
Did you know? Quiz
Information on different types of hypnotics: benzodiazepines and z-drugs
Temporary advantages: sleep and relaxation
Negative side effects: sleep, drowsiness, cognitive effects, behaviour and emotion, social context, risk of traffic accidents, fall risk, financial context, tolerance and dependence, other remarks.
Tolerance and dependence
What is it?
Test yourself
About withdrawal
Summary: core message

Chapter 3
True or not? Quiz
Better solutions

Chapter 4
Info + checklist on sleeping habits
Results of the checklist

Chapter 5
Info on sleep hygiene and discontinuation of hypnotics
Sleep hygiene tips
Cognitive component info
5G-exercise
Stimulus control info

Chapter 6
The challenge: brief questionnaire to map motivation
Balance tool
Visual result of balance tool
Info about tapering
Tapering schedule: to be completed by the GP

3. Participants information

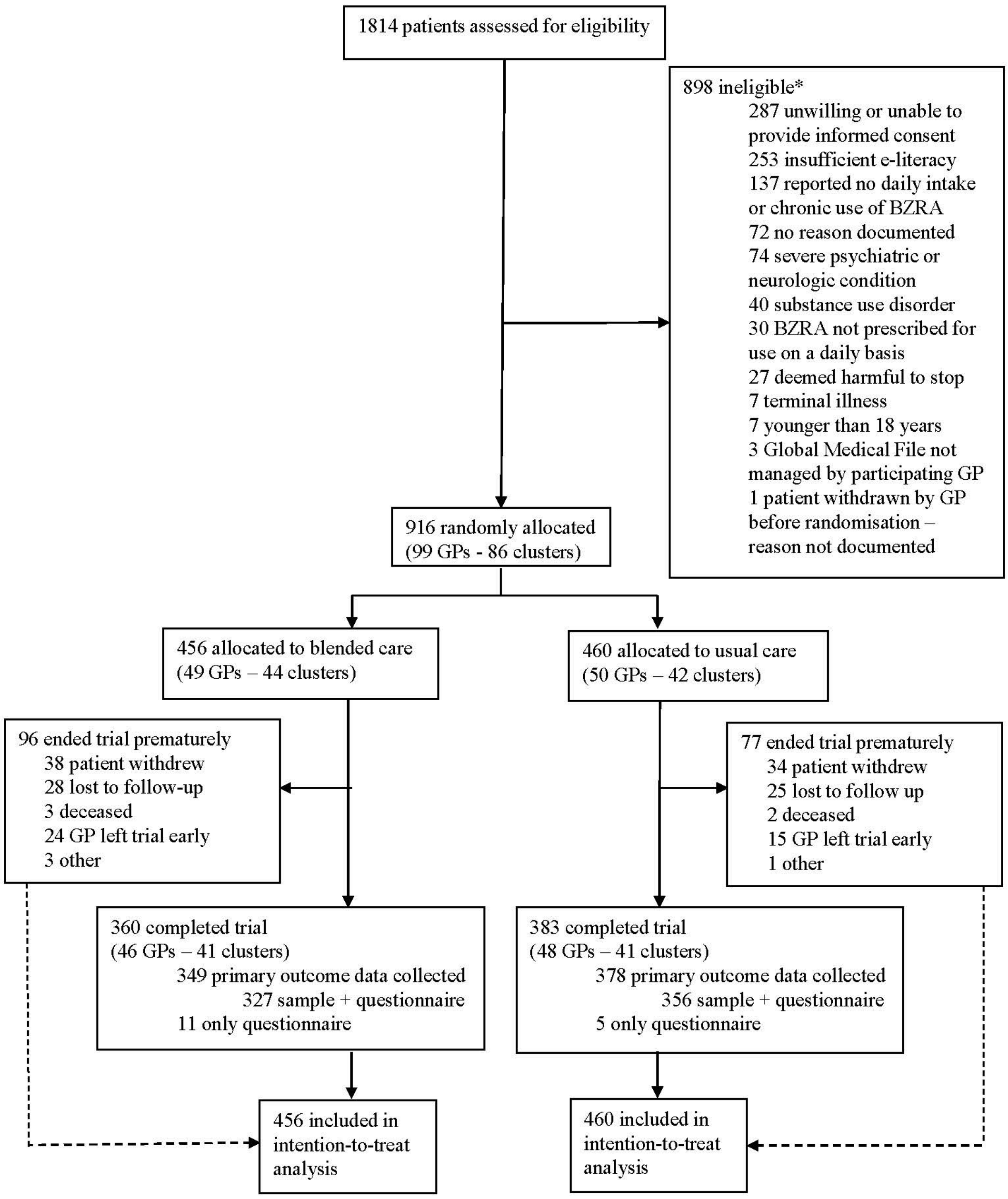

*Figure 2. Flowchart diagram of patient participation. *Patients could be deemed ineligible based on more than one in- or exclusion criterium. Primary outcome data = urine sample at week 52. Adapted from Coteur et al. 2022* [18]

*Table 1. Sociodemographic data of participants in interviews (patients) and focus groups (general practitioners). *There were 8 interactive elements available which could be completed multiple times. Cut-off was set at 5. **N represents the number of surgeries in this category from the intervention group.*

	**Patient-participants (N=18)**
Age in years, mean (SD)	56.22 (11.61)
Sex: female, n (%)	15 (83%)
Duration of BZRA use, mean (SD)	10.33 (7.30)
Discontinuation status, n (%)	
Yes, full stop	7 (39%)
No, reduced	2 (11%)
No, stable	7 (39%)
No, increased	2 (11%)
Intense use of intervention*	
Completed 5 exercises or more, n (%)	11 (61%)
Completed no exercises, n (%)	2 (11%)
	**General practitioner-participants (N=19)**
Sex: female, n (%)	11 (58%)
Language: Dutch, n (%)	14 (74%)
Surgery category, n (%)	
Solo (N=7)**	2 (11%)
Group	
Unidisciplinary (N=21)**	10 (53%)
Multidisciplinary (N=10)**	3 (16%)
Community health center (N=11)**	4 (21%)

*Table 2. Baseline characteristics of 916 randomized patients (*The 3 most prevalent co-morbidities are listed. The category 'other' includes asthma, ischemic heart disease, COPD, cancer, cerebrovascular disorders, heart failure, and rheumatoid arthritis.) Adapted from Coteur et al. 2022* [18]

	**Blended care (n=456)**	**Usual care (n=460)**
Age, years	61.72 (11.57)	61.10 (11.44)
Missing data	2 (<1%)	0
Sex		
Female	329 (72%)	324 (70%)
Male	124 (27%)	135 (29%)
X#	3 (<1%)	1 (<1%)
Missing data	0	0
Co-morbidity*		
None registered	98 (21%)	142 (31%)
Depression	115 (25%)	135 (29%)
Diabetes	43 (9%)	43 (9%)
Migraine	25 (5%)	21 (5%)
Other	133 (29%)	87 (19%)
Missing data	42 (9%)	32 (7%)
# BZRA used		
0 BZRA	14 (3%)	18 (4%)
1 BZRA	339 (74%)	336 (73%)
2 BZRA	53 (12%)	65 (14%)
3 BZRA	5 (1%)	8 (2%)
4 BZRA	1 (<1%)	0
Missing data	44 (10%)	33 (7%)
Defined Daily Dose		
Mg diazepam (average (SD))	10.10 (8.95)	10.83 (8.84)
Range, mg	0-110	0-70
Missing data	44 (10%)	33 (7%)

#X refers both to patients who did not want their sex to be registered by the GP, and to intersex

4. COM-B and TDF

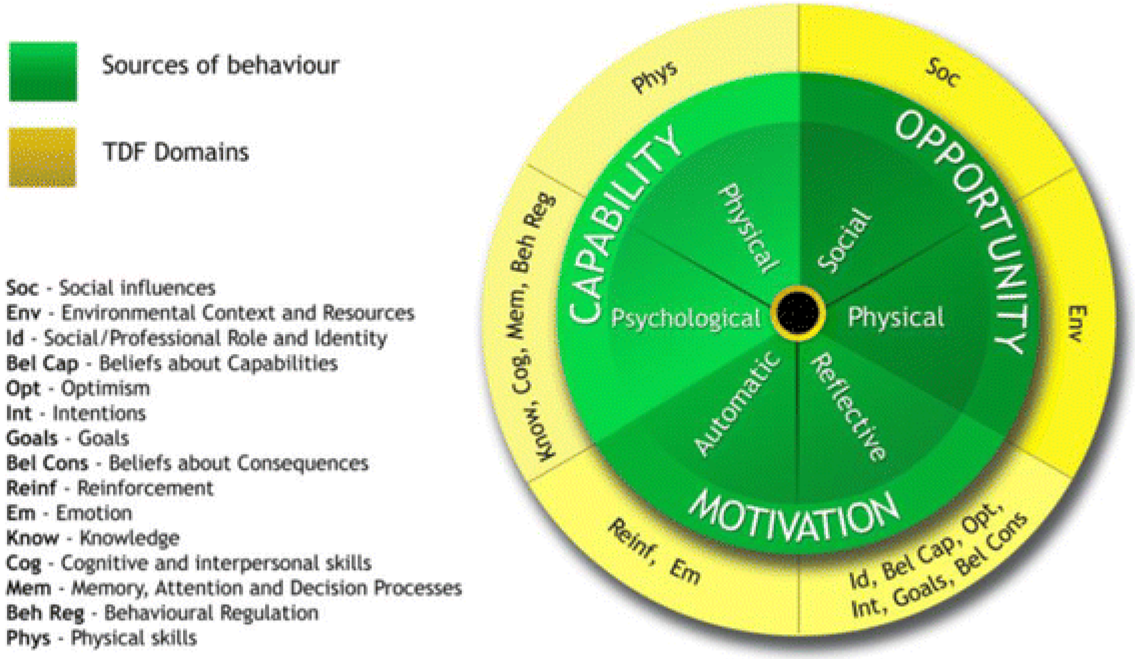

Figure 3. COM-B model and associated domains from the Theoretical Domains Framework by Atkins. et al 2017 [19]

5. Questionnaire and interview guides

The following questionnaire and interview guides were translated from Dutch to English with DeepL Translator on 1 February, 2023.

5A. Questionnaire to survey GPs about recruitment

In the following questions, by patients we always mean those with primary insomnia and chronic use of z-drugs and/or benzodiazepines ((z-)BZD).

What is your situation?

- I was able to recruit enough patients and am actively participating in the study.
- My participation in the study was terminated early because of insufficient patients.
- My participation in the study was stopped early because of other reasons.

During the recruitment period (2019), I recall having invited approximately the following number of patients to participate in the Big Bird study: ___.

Did this recruitment increase your daily workload?

- Yes
- No
- Other: ___________

What reasons do you see for this increase in workload? ___________

Before signing my contract from the Big Bird study, I conducted an audit. This with the intention of assessing whether sufficient patients with primary insomnia and chronic use of (z-)BZD are being treated in my surgery.

- Yes
- No
- Other: ________

I had initially planned to actively approach patients eligible for the Big Bird study.
Eg. send stop letter or call and invite them

Continuous scale with markers strongly disagree – neutral - strongly agree

I saw patients during the recruitment period who would be eligible for the study. E.g., during other consultations

- Yes
- No
- Other: _________

I approached patients during the recruitment period to participate in the Big Bird study. Eg. during others consultations or by calling them.

- Yes
- No
- Other: _________

I remember an e-poster or other recruitment material for the Big Bird study being used in the waiting room.

- Yes
- No
- Other: _________

During the recruitment period, I forgot to approach an important part of the patients to participate in the Big Bird study.

Continuous scale with markers strongly disagree – neutral - strongly agree

During the recruitment period, I deliberately did not approach some of the patients because I suspected they were there not open to participation.

Continuous scale with markers strongly disagree – neutral - strongly agree

Most of the patients I approached during the recruitment period to participate in the Big Bird study was not interested.

Continuous scale with markers strongly disagree – neutral - strongly agree

In my opinion, a possible lack of interest on the part of patients is mainly due to:

- ☐ Their lack of computer skills.
- ☐ The subject of the study: (z-)BZD and sleep.
- ☐ The way the study was explained, and the fear that they would have to taper off their sleep medication.
- ☐ The time investment (of the patient).
- ☐ Uncertainty about the purpose of the study.
- ☐ Uncertainty about their contribution to the research.
- ☐ Other: ___________

Your comments regarding the recruitment process for the Big Bird study and your experience during this period are an added value for this and future research. Specifically: could the research team have done more to support you in recruiting patients for this study? Please provide your feedback below: ___________

5B. interview guides

Table 1. Interview guide for the asynchronous focus groups with GPs, using the platform Focusgroupit

Motivation	What was the main reason for you to participate in the Big Bird study?
Experience	Does the study meet your reason for participating?
	What is your overall experience with the study?
Blended care	What does blended care mean to you?
	What experience do you have with blended care? Please include previous experience (before the Big Bird study).
Added value	In your opinion, what added value does blended care offer over usual care in phasing out benzodiazepines and z-drugs?
In practice	How have you used the *Sleep Care Free* self-study modules?
Retaining *Sleep Carefree*	What should definitely be retained from the self-study modules?
	What aspects did you find useful?
Changes *Sleep Carefree*	What would you like to change about the self-study modules?
	What aspects did you find less useful in practice?
Relationship with the patient	In what ways did offering blended care change your consultations regarding benzodiazepines and z-drugs?
	What impact did you notice on follow-up with patients?
Patient motivation	What do you think was the main reason your patients wanted to participate in the study?
	Were certain profiles/groups of individuals more difficult to motivate? Which ones and why?
COVID-19	How did the COVID-19 situation (affect) the conduct of the study in your practice?
Also...	Thank you for your contribution in both the Big Bird study and this process evaluation. You may always return to the topics and respond to what your colleagues have noted.
	Would you have liked to share anything else?

Interview guide for interviews with current and past BZRA users

Introduction and ICF verification

Personal information (ask if not already known)
- Family situation
- Place of residence/postal code
- Employment situation (what do you do in daily life?)

Part 1 - context
- Since when have you been taking sleep medication? How long have you been taking this medication?
- How did you come to start this medication? What was the reason?
- On whose initiative did you start then (PROBE: on doctor's recommendation, own direction, friends/acquaintances?)
- If by doctor: what did you think of the doctor's recommendation? What other solutions, if any, did the doctor suggest?
- What advice did the (family) physician give you when prescribing? (PROBE: difference first prescription and repeat prescriptions, dosage, use, possible side effects, prescribed duration of use)

Current BZRA users:
- How important is the use of this medication to you? (PROBE: would you describe yourself as a satisfied user?)
- Have you ever attempted to reduce or stop?
- What difference does using sleep medication make to you?
- Do you have a particular bedtime routine? What does it consist of?

Past BZRA users:
- How important was the use of this medication to you? (PROBE: would you describe yourself as a satisfied user?)
- What difference did using sleep medication make to you?
- What prompted you to stop?
- How did you approach this? (PROBE: phasing out, stopping all at once, previous attempts)
- Did you experience any particular symptoms during the phaseout? Which ones?
- Did you experience any particular symptoms after quitting? Which ones?
- Did you experience support from your environment while quitting? Why?
- Have you ever tried quitting or tapering in the past?

- What do your days/nights look like now without this medication?
- Do you have a particular bedtime routine? Has it changed since you stopped taking medication?

- Are you currently using other treatments for your sleep problems? If so, which ones? (PROBE: psychotherapy, natural products, yoga, relaxation, etc.)

Part 2 - the intervention
- Did your doctor explain that an online intervention was available?
- How often (approximately) did you use the online self-help modules (Sleep Carefree)?
    - On whose initiative did you use these modules? (PROBE: Did the doctor encourage you? Were you curious? Did your partner or children recommend that you check it out?)
- What was it like for you to get regular emails about it?
- How did you experience using the online self-help modules on Sleep Carefree?
- In what ways has using Sleep Care Free had an impact on you?
- What changes have occurred in your thinking as a result of participating in this study? (PROBE: Has your opinion about the use of sleep medication changed? In what ways?)
- To what can these changes be attributed? (PROBE: how did these changes come about? Share of the physician? Share of yourself? Share of the website?)
- In what ways do these new thoughts affect your use of sleep medication? (PROBE: how has this affected your routine?)

- What information do you remember from Sleep Carefree? (PROBE: Information about sleep architecture? Information about the medication? Quiz? Sleep hygiene tips? Balance assignment? Sleep diary?) cfr. Screenshots
- Did your doctor complete a phase-out schedule (in the online modules)?
    - How was it working with that? Tips for improvement?
- What did you find helpful about these online self-help modules? (PROBE: What did you find helpful about this section? Success experience?)

- What did you find useless about these online self-help modules? (PROBE: What did you find useless about this component? Difficulty experienced?)
- How do you think we can improve the modules?
- If the self-help modules were available to everyone for free, would you continue to use Sleep Carefree? (PROBE: why yes/no? What would motivate you to use the online modules?)

Part 3 - blended care

- What do you think about the blended approach in this study (PROBE: online information and consultation with GP?)?
    - How was the tool used during the consultation?
- How would you describe your relationship with your (family) physician? (PROBE: is this relationship evolving? Differences from before? What could be better?)
- Did your participation in this study impact your relationship with your physician? If so, what ran differently? (PROBE: Did the doctor give different information? Was the doctor seen more? Did you feel different as a patient? E.g., more knowledge about the medication, which led to different conversations?)
- How do you feel about this? Are these lasting changes in your opinion?

- What aspects of participating in this study had the most impact on you?
- What made you decide to participate in this study?
- What expectations did you have from your participation in this study? In what ways were they met or not met?
- Was it a barrier for you that the information was offered as a website?
- If it was presented in a different way, would you use it more? In an app, on paper, etc.
- Are there any other barriers you have experienced? What made you not use it yourself?
- What would be ideal for you? How do you prefer to receive information?

Part 4 – future

Current BZRA users:

- Would you like to change something about your sleep medication use in the future?
- What would you need in order to do this?
- What do you think it would take to stop this medication completely at some point?

Past BZRA users:

- You have stopped taking sleep medication, congratulations. How satisfied are you with this?
- What do you need to keep this up? (PROBE: what adjustments in your daily life do you consider necessary? What motivates you to continue without medication? How confident do you feel in continuing without medication?)
- Suppose you were sleeping poorly again for 2-3 weeks. How would you handle that?

Part 5 - impact of COVID-19

- 2020 is an exceptional year because of the global COVID-19 situation. In what ways is COVID-19 affecting your life?
- What impact does this situation have on your sleep?

Current BZRA users:

- Has your medication use been adjusted in recent months? If yes, in what way?

Concluding question

- Are there any other things you would like to pass along?
